# N4 DNA recognition by STAT6: structural and functional implications

**DOI:** 10.1007/s13238-017-0380-z

**Published:** 2017-02-20

**Authors:** Xiang Zhou, Zhengfan Jiang

**Affiliations:** 1 Key Laboratory of Cell Proliferation and Differentiation of the Ministry of Education, State Key Laboratory of Protein and Plant Gene Research, School of Life Sciences, Peking University, Beijing, 100871 China; 2 Peking-Tsinghua Center for Life Sciences, Beijing, 100871 China

On Nov. 1st 2016, a research paper titled *Structural basis for DNA recognition by STAT6* was published online on *PNAS* (Li et al., 2016), which revealed for the first time the mechanism of signal transducer and activator of transcription 6 (STAT6) recognition by DNA with N4 site. The authors are from the Institute of Biophysics, Chinese Academy of Sciences (CAS).

Virus infection is omnipresent, while most of the hidden hazard is neglected in the early phase of infection. An example is the herpes simplex virus, which can cause acute or chronic infectious diseases, such as viral hepatitis, AIDS, the recent Ebola hemorrhagic fever in West Africa, and so on. Accurate and sophisticated antiviral immunity has been established in long-term evolution from the interplay of the virus and the host. Innate immunity is the first line of defense against viral infection as surveillance of a variety of invading pathogens. Activation of the immune response will lead to the expression of a series of antiviral genes, which ultimately fight and clear the infection. Understanding the molecular basis of innate immunity is thus very important for the treatment of viral diseases.

 Germline-encoded pattern recognition receptors (PRRs), like Toll-like receptors (TLRs), RIG-I-like receptors (RLRs) and NOD-like receptors (NLRs), recognize conserved molecular structures known as pathogen-associated molecular patterns (PAMPs) and initiate host antimicrobial response such as the production of type I interferons and other cytokines (Takeuchi and Akira, 2010). DNA is normally found in the nucleus of the cell. Localization of DNA to the cytosol is associated with tumorigenesis or viral infection. Many DNA sensors (such as AIM2, RNA polymerase III, LRRFIP1, IFI16, DDX41, DNA-PKC, Ku70, DHX36/DHX9, DHX36/DDX1/DDX21, cGAS etc.) and adaptor molecules (such as STING/ERIS/MITA, IPS-1/MAVS/VISA/Cardif, NLRC3 etc.) in the signaling pathway have been identified in recent years. A lot of structure-function studies have been done on these molecules (Ouyang et al., 2012; Parvatiyar et al., 2012; Ru et al., 2013; Shaw et al., 2013; Zhang et al., 2014; Zhao et al., 2014; Jiang et al., 2016; Ni et al., 2016). The cGAS-STING pathway is a component of the innate immune system that functions to detect the presence of cytosolic DNA and, in response, trigger expression of inflammatory genes and acts to detect cytosolic DNA and induce an immune response (Ma and Damania, 2016).

In 2011, through the functional screening of cDNA expression library, Dr. Jiang’s lab in Peking University discovered that the STING-TBK1 axis can activate the transcription factor STAT6, which plays an important role both in adaptive immunity and in the antiviral innate immunity. The study reveals the molecular mechanism of the activation of transcription factor STAT6 by STING, from molecules, cells, and mice. Their results provide a new role of STING-mediated host innate immune responses, and offer a new strategy for the treatment of the autoimmune diseases caused by viral infection (Chen et al., 2011; Chen and Jiang, 2013).

The signal pathway of STING has been the focus of attention for researchers, and STAT6 has attracted researchers’ attention because of its interaction with STING. STAT6 was previously reported to be a member of the signal transducers and activators of transcriptions (STATs), involved in the JAK-STAT signaling pathway, which is regulated by IL-4/IL-13. Signaling from the cell membrane, STAT6 is activated and transferred to the nucleus to stimulate the transcription of a series of downstream genes, which plays a key role in Th2 cell differentiation. The expression of STAT6 is closely related to inflammation and a variety of tumors, thus it may become a new marker for tumor diagnoses. With important clinical significance, STAT6 can be used as a prognostic indicator and a new target for treatment. STAT6 as a transcription factor in the cytoplasm can form dimers upon phosphorylation. The STAT6 dimers then translocate into the nucleus and bind to the target gene promoter regions to initiate a series of gene expression. The STAT family proteins recognize similar DNA sequences, the basic structure of which is TTCN_3/4_GAA, but the intermediate spacer length for the DNA palindromic sequence is different for diverse STAT proteins. In the mammalian STAT family, STAT6 is the only member of the family that recognizes the N4 locus promoter (TTCN_4_GAA), while the other STATs are predominantly recognizing the N3 site promoter (TTCN_3_GAA). The mechanism of DNA recognition for STAT6 is a critical question which has not been solved in the past decades.

In current study (Li et al., 2016), the structure of the phosphorylated dimer of the STAT6 core region was analyzed by structural and cellular biology method. At the same time, the precise three-dimensional structure of a homodimer of phosphorylated STAT6 core fragment (STAT6^CF^) alone and bound with the N3 and N4 DNAs were displayed, which has never been studied before. Therefore, the researchers have collected sufficient structural resources for in-depth structure analysis. The structure of phosphorylated STAT6^CF^ used a similar mechanism to form a “V” shape with other STAT proteins studied. It was found that the STAT6 dimer produced a significant conformational change after binding to nucleic acid, and the position of the key amino acid H415 was changed in the direction of DNA double helix axis after N3 and N4 DNA binding, the distance (4 Å) close to the vertical distance between two adjacent bases in DNA double helix (approximately 3.4 Å Rise/bp along the axis of a B-DNA double helix). This work shows for the first time the conformational change of DNA before and after binding to the STAT family protein. Molecular dynamics simulations and small-angle X-ray scattering experiments (SAXS) showed that the conformation of STAT6 is more stable after DNA binding. Compared with the other reported STAT proteins, phosphorylated STAT6 dimer has a “V” shape with larger angle than other STAT proteins, resulting in a more dynamic range between the two molecules in the dimer structure. At the same time, what caused the researchers’ attention was the finding that H415 on STAT6 is the only amino acid that directly interacts with the DNA. Besides STAT5, it was found that the amino acid corresponding to H415 was N (Asn) in other STAT proteins. *In vitro* experiments showed that the ability to recognize N4 site DNA decreased after H415 was mutated to N and the ability to recognize N3 site DNA increased, while the ability of DNA binding by STAT1 was decreased for N3 DNA but increased for N4 DNA by the N460H mutation. Cellular experiments also demonstrated that STAT6 H415 plays a key role in STAT6-specific N4 DNA binding. The reason that STAT5 can not identify N4 DNA may be because on the one hand, STAT5 dimer angle is slightly smaller, and on the other hand, the stretching direction of H471 side chain is different from that of STAT6 H415.
